# A phosphohistidine phosphatase promotes starvation survival by dephosphorylating nucleoside diphosphate kinase

**DOI:** 10.1016/j.celrep.2025.116813

**Published:** 2026-01-12

**Authors:** Akash R. Sinha, Mark Goulian

**Affiliations:** 1 Department of Biology, University of Pennsylvania, Philadelphia, PA 19104, USA; 2 Department of Physics & Astronomy, University of Pennsylvania, Philadelphia, PA 19104, USA; 3 Lead contact

## Abstract

Nucleoside diphosphate kinase (Ndk) is a ubiquitous enzyme that maintains the cellular nucleoside triphosphate (NTP) pool and participates in many other pathways of eukaryotes and prokaryotes. Here, we show that in *Escherichia coli*, Ndk is regulated by dephosphorylation of its phosphohistidine intermediate via the phosphatase SixA, thereby inhibiting nucleotide phosphoryl transfer activity. We further show that loss of this regulation alters the metabolic state of *E. coli* in low-nutrient conditions and reduces survival in long-term stationary phase. Similar regulation of Ndk by a phosphohistidine phosphatase has been reported previously for human cells, although the molecular interactions differ. The prevalence of SixA and Ndk orthologs in prokaryotes and the appearance of this regulatory mechanism in both *E. coli* and humans suggest that phosphohistidine phosphatase-mediated control of nucleoside diphosphate kinases may be widespread.

## In brief

Sinha and Goulian demonstrate that the phosphatase SixA is critical for *E. coli* to survive the starvation stress of long-term stationary phase. Genetic and biochemical analyses show that this survival advantage depends on SixA-mediated dephosphorylation of nucleoside diphosphate kinase, revealing a new mechanism for regulating this enzyme in bacteria.

## Graphical Abstract

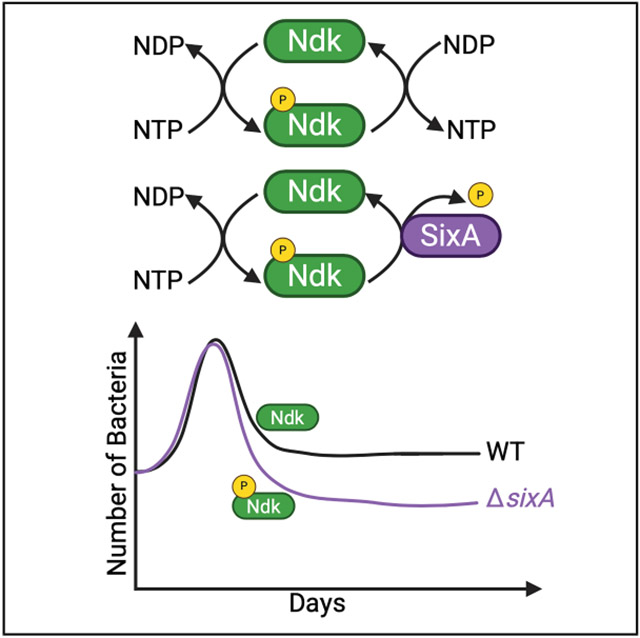

## INTRODUCTION

Nucleoside diphosphate kinase (Ndk) catalyzes the transfer of a phosphoryl group from a nucleoside triphosphate to a nucleoside diphosphate, an activity that maintains nucleotide homeostasis within cells.^1-4^ Perhaps for this reason, the enzyme is found in virtually every organism. One of Ndk’s key features is its ping-pong catalytic mechanism: a nucleoside triphosphate (NTP) first phosphorylates the enzyme to produce a phosphohistidine intermediate, the resulting nucleoside diphosphate (NDP) is released, and the enzyme then transfers the phosphoryl group from phosphohistidine to a second NDP to produce an NTP.^5-7^ Ndk is not essential in many organisms, which reflects the existence of other pathways to phosphorylate NDPs.^4,8,9^ Nevertheless, even when alternative pathways are present, eliminating Ndk is not without consequences. For example, in *E. coli*, an *ndk* deletion has a mutator phenotype^9^ and is also more resistant to some nucleoside analogs.^10-12^ Nucleoside diphosphate kinase is also known to have other functions in addition to maintaining the nucleotide pool. In prokaryotes, Ndk has been reported to be involved in cell division, quorum sensing, and virulence.^13-18^ In eukaryotes, the enzyme has multiple isoforms, some of which have protein kinase or other enzyme activities and participate in diverse pathways, such as membrane remodeling, G protein activation, ion channel regulation, metastasis suppression, and pro-tumorigenic activities.^1,3,19-22^

Given Ndk’s role in NDP phosphorylation and other processes, it is not surprising that its activity is modulated. In bacteria and eukaryotes, Ndk is regulated transcriptionally.^23,24^ In addition, in eukaryotes, post-transcriptional regulation has been described.^3,21,25^ One notable example that is relevant to this study is the regulation of the human nucleoside diphosphate kinase NDPK-B by PGAM5, a phosphatase that acts on the NDPK-B phosphohistidine intermediate.^25^ Thus, PGAM5 functions as a phosphohistidine phosphatase.

To date, relatively few enzymes have been shown to function as phosphohistidine phosphatases *in vivo*. The first example to be identified was SixA, which is found in *Escherichia coli* (*E. coli*) and many other bacteria.^26,27^ SixA was discovered due to its ability to dephosphorylate the *E. coli* sensor kinase ArcB,^27,28^ although the significance of this interaction *in vivo* is unclear.^29,30^ Subsequently, the enzyme was shown to dephosphorylate the phosphocarrier NPr of the nitrogen-related phosphotransferase system (PTS^Ntr^) and to affect potassium homeostasis in *E. coli*.^30-33^ However, SixA orthologs are present in diverse bacterial species, some of which lack phosphocarrier proteins,^26,32^ suggesting there might be additional targets for this enzyme. Indeed, it was recently shown that SixA also dephosphorylates the glycolytic enzyme PfkA,^34^ which is found in many but not all bacteria that have SixA. Thus, the full range of SixA’s targets remains to be explored.

Here, we report that in *E. coli,* SixA also dephosphorylates Ndk *in vitro* and *in vivo*. We further show that the loss of SixA reduces *E. coli* survival in long-term stationary phase in an Ndk-dependent manner and that the resulting Ndk dysregulation alters the metabolic state of starving cells. Given the presence of this regulatory mechanism in both humans and *E. coli*, and the cooccurrence of SixA and Ndk orthologs in other bacterial phyla, the regulation of nucleoside diphosphate kinase by phosphohistidine phosphatases may be widespread in bacteria and other organisms as well.

## RESULTS

### A *sixA* deletion has a fitness defect in prolonged stationary phase

**Figure 1.**
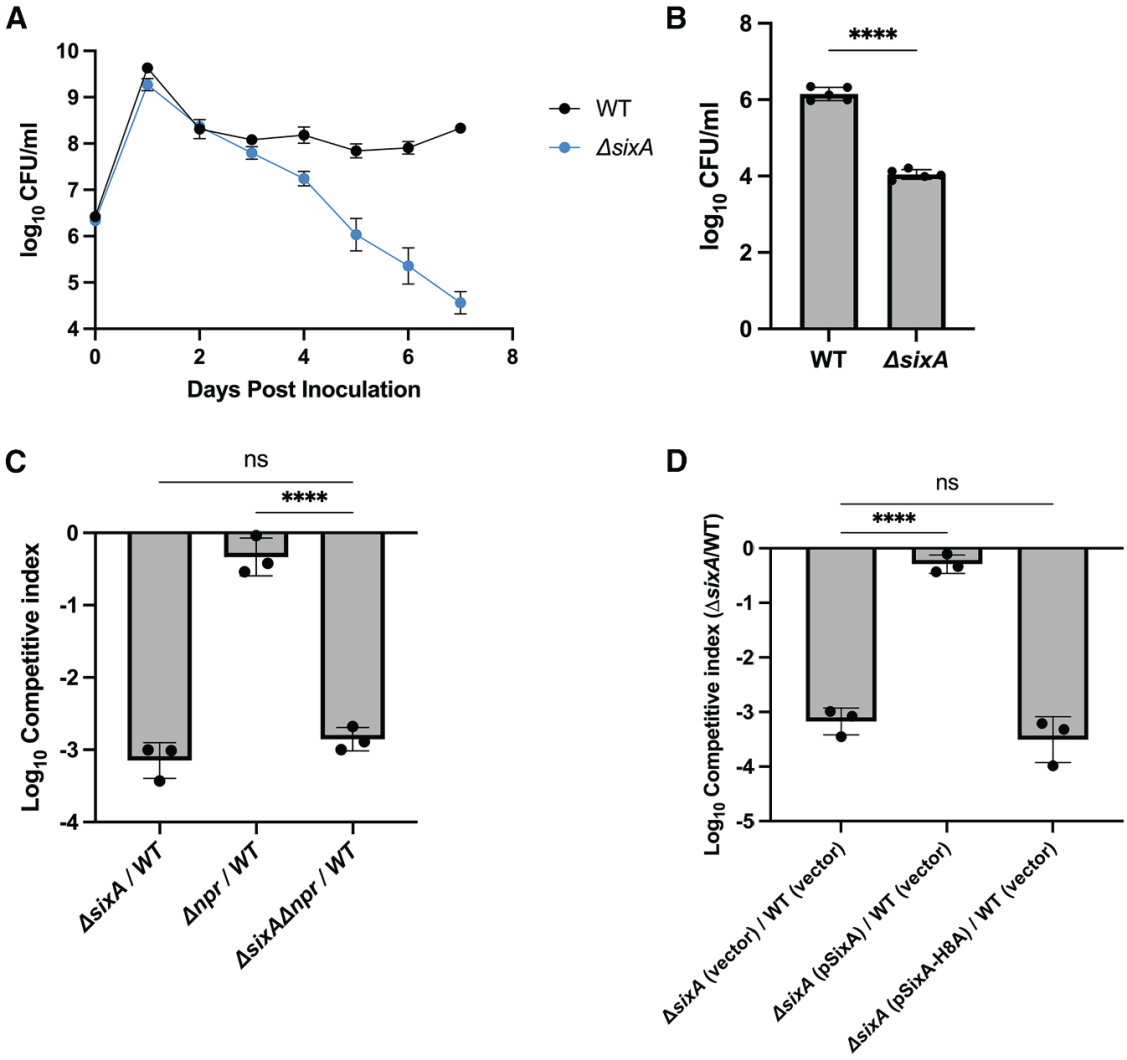
*ΔsixA E. coli* has a survival defect in long-term stationary phase (A) Seven-day co-culture of wild type (WT) and *ΔsixA* in LB. (B) Fourteen-day monocultures of wild type and *ΔsixA* in LB. (C) Seven-day co-culture of wild type with the indicated mutants in LB. Competitive index = (CFU[day 7]_mutant_/CFU[day 0]_mutant_)/(CFU[day 7]_WT_/CFU[day 0]_WT_). (D) Complementation of the *ΔsixA* survival defect in seven-day co-cultures in LB with 50 μg/mL ampicillin. Error bars denote standard deviations. Symbols are the averages of triplicates in (A) and denote replicates in (B)–(D). Filled bars in (B)–(D) denote averages. Statistical significance was calculated by an unpaired *t* test (B) or Dunnett’s multiple comparison test (C and D). ns, **, ***, and **** represent *p* values > 0.05, ≤ 0.01, ≤ 0.001, and ≤ 0.0001, respectively. The data shown here are representative of at least three independent experiments. Strains used in the experiments are listed in Table S1.

While looking for phenotypes of a SixA^−^ strain (*ΔsixA*) in different growth conditions, we noticed a strong fitness defect in long-term stationary phase in lysogeny broth (LB). In seven-day competitions with wild type, *ΔsixA* began to show a relative survival defect by day three, and by day seven, its colony-forming units (CFUs) had declined by 10^3^–10^4^ (Figure 1A). Monocultures showed a similar decline, albeit at a slower rate, with *ΔsixA* CFU 100-fold lower than those of wild type by day 14 (Figure 1B). The stronger defect of *ΔsixA* when in competition with wild type could be a result of the increased rate of nutrient depletion from the media in these conditions, relative to monoculture.

To determine if this phenotype depends on NPr, the previously identified SixA target, we competed *Δnpr* and the double deletion *ΔsixA Δnpr* separately against wild type. The *ΔsixA Δnpr* and *ΔsixA* strains had similar defects, whereas *Δnpr* behaved similarly to wild type (Figure 1C). In addition, the survival defect of the *ΔsixA* strain was eliminated by introducing a plasmid expressing SixA, but not with a plasmid expressing a catalytically inactive SixA mutant (SixA H8A)^27,30^ (Figure 1D). These results indicate that SixA phosphatase activity is important for *E. coli* survival in long-term stationary phase cultures through a mechanism that is independent of NPr.

### The *ΔsixA* fitness defect is suppressed by *cytR* and *ndk* deletions

**Figure 2.**
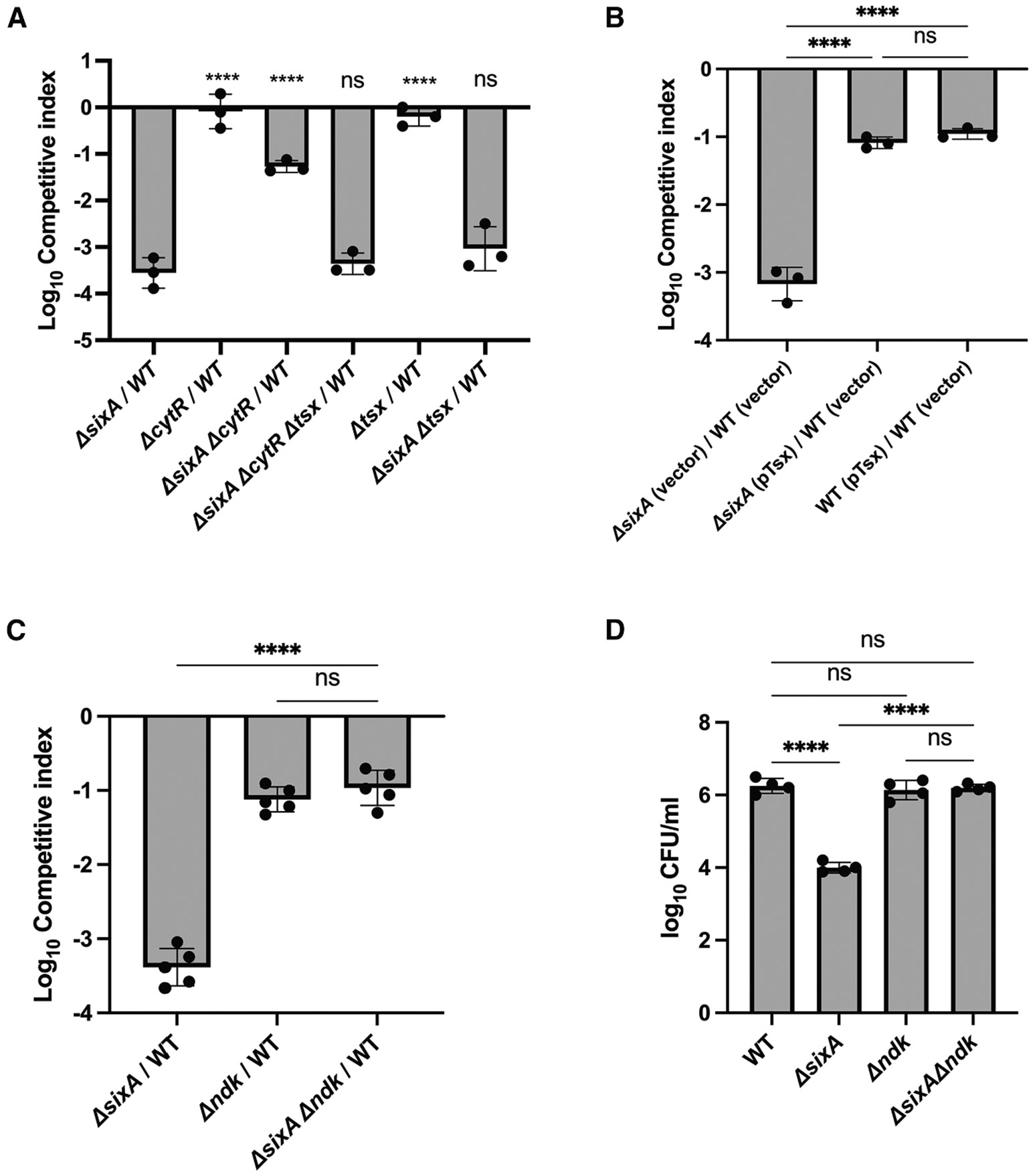
Genes associated with nucleoside metabolism suppress the *ΔsixA* fitness defect (A–C) Seven-day co-culture of wild type with the indicated strains in LB (A and C) or LB with 50 μg/mL ampicillin (B). (D) Fourteen-day monocultures of the indicated strains in LB. Symbols, error bars, and filled bars denote replicates, standard deviations, and averages, respectively. Statistical significance was calculated using Dunnett’s test for multiple comparisons against *ΔsixA*/WT (A) or against *ΔsixA Δndk*/WT (C) and Tukey’s multiple comparison test (B and D). ns, **, ***, and **** represent *p* values > 0.05, ≤ 0.01, ≤ 0.001, and ≤ 0.0001, respectively. The data shown here are representative of at least three independent experiments. Strains used in the experiments are listed in Table S1.

To explore how SixA affects long-term stationary phase survival, we mutagenized the *ΔsixA* strain with a transposon and selected for mutants that showed increased fitness in competitions with wild type (see method details). Transposon mutagenesis was used because the insertions can be readily transferred to clean strain backgrounds by phage transduction to eliminate spontaneous mutants that increase stationary phase fitness independently of *sixA*. From the selection, we isolated mutants with transposon insertions in the gene *cytR*. We subsequently confirmed that introducing a *cytR* deletion into the *ΔsixA* strain partially suppressed the fitness defect associated with the loss of *sixA* (Figure 2A). In addition, a *cytR* deletion had no effect on fitness when introduced into a wild-type (SixA^+^) strain. CytR is a transcription factor that regulates genes involved in nucleoside uptake and metabolism.^35-37^ One of the genes repressed by CytR, *tsx,* encodes a nucleoside-specific porin in the outer membrane that is important for the uptake of nucleosides at low concentrations.^38,39^ Deleting *tsx* in a *ΔsixA ΔcytR* strain restored stationary phase survival to a level similar to that of *ΔsixA*, but the *tsx* deletion had no effect on survival in CytR^+^ backgrounds (Figure 2A). In addition, the introduction of a Tsx expression plasmid into the *ΔsixA* strain showed an increase in fitness similar to that produced by *ΔcytR* (Figure 2B). Although the suppression caused by the Tsx expression plasmid was partial, the fitness defect that remained was comparable to that of the wild-type strain containing the plasmid. This residual defect may reflect a fitness cost associated with high-level expression of Tsx. Taken together, these results suggest that *ΔcytR* suppresses the survival defect of the *ΔsixA* strain by increasing nucleoside uptake and, therefore, that SixA might have a role in nucleoside metabolism.

As discussed above, the phosphatase PGAM5 dephosphorylates the phosphohistidine intermediate of the nucleoside diphosphate kinase NDPKB in humans. Given our results suggesting a role of SixA in nucleoside metabolism, we considered the possibility that the *E. coli* nucleoside diphosphate kinase, Ndk, is a SixA substrate and that the loss of this Ndk dephosphorylation mechanism accounts for the long-term stationary phase fitness defect of a *ΔsixA* strain. To test this hypothesis, we compared the fitness of *ΔsixA*, *Δndk*, and *ΔsixA Δndk* in seven-day competitions against wild type. The competitive indices of *ΔsixA Δndk* and *Δndk* were comparable and roughly 100-fold higher than that of *ΔsixA* (Figure 2C). Furthermore, we observed similar results in 14-day monocultures (Figure 2D), although in this case, *Δndk* and *ΔsixA Δndk* did not produce a detectable decrease in CFU relative to wild type. Thus, with respect to stationary phase survival, *Δndk* suppresses the fitness defect of *ΔsixA.* In addition, the fact that the fitness defects of *ΔsixA Δndk* and *Δndk* are comparable is consistent with the hypothesis that SixA dephosphorylates Ndk.

### SixA affects Ndk-associated phenotypes

**Figure 3.**
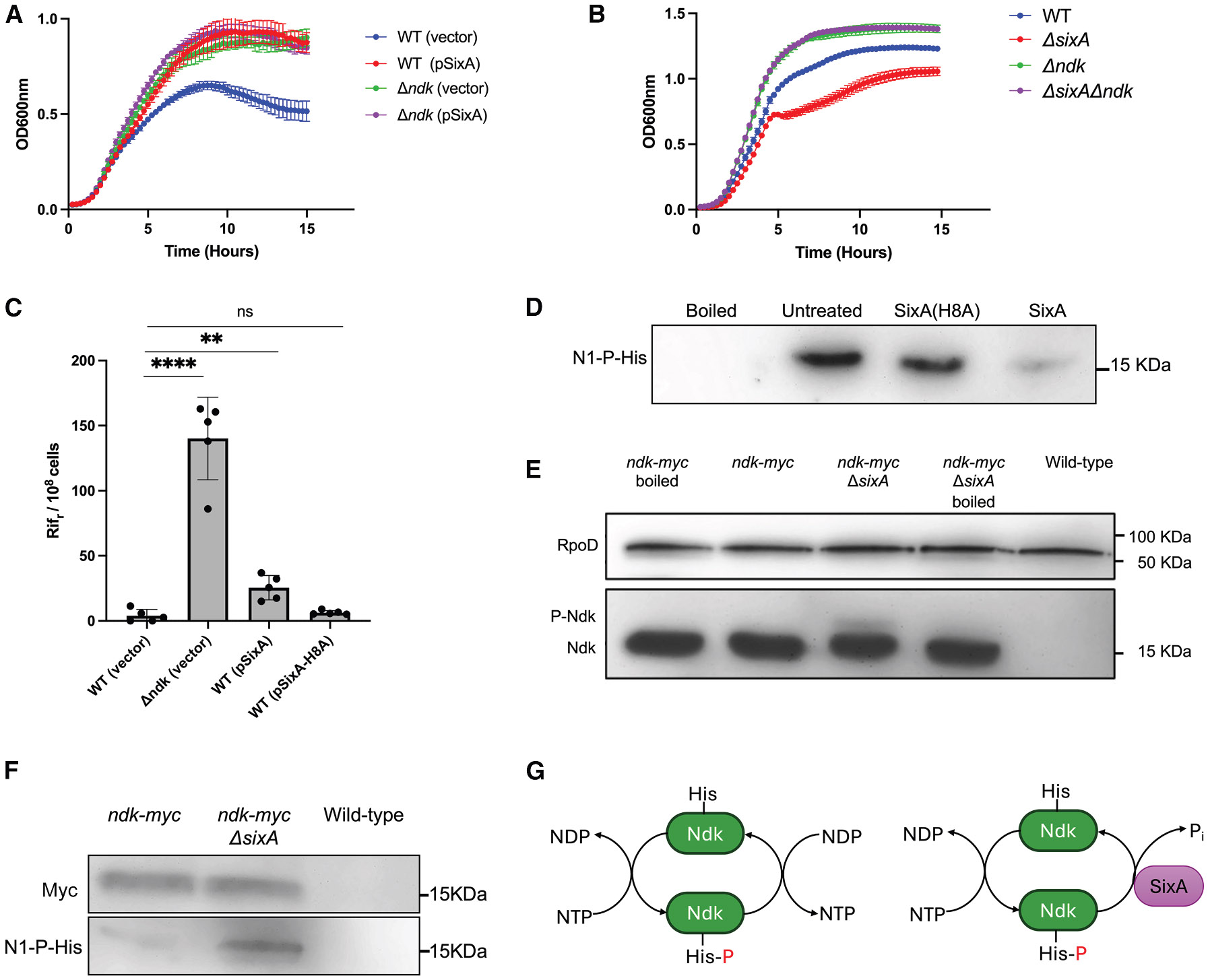
SixA dephosphorylates Ndk *in vitro* and *in vivo* (A) Growth curves of wild-type or *Δndk E. coli* containing a plasmid expressing SixA (pSixA) or an empty vector in LB containing 20 ng/mL azidothymidine (AZT) and 50 μg/mL ampicillin. Symbols are the averages over three wells in a 96-well plate, and error bars are the standard deviations. (B) Growth curves of the indicated strains in LB containing 10 ng/mL AZT. Symbols and error bars are as in (A). (C) The number of rifampicin-resistant cells per 10^8^ cells in LB overnight cultures of the indicated strains. The symbols, filled bars, and error bars are replicates, averages, and standard deviations, respectively. Statistical significance was calculated by Dunnett’s multiple comparison test. ns, **, ***, and **** represent *p* values > 0.05, ≤ 0.01, ≤ 0.001, and ≤ 0.0001, respectively. (D) Western blot of *in vitro* reactions containing phosphorylated Ndk with either no addition, SixA, or the catalytically inactive mutant SixA(H8A). Phosphorylated protein was visualized with anti-N1-phosphohistidine antibody. (E) Western blot from a Phos-tag acrylamide gel showing relative levels of Ndk phosphorylation *in vivo*. RpoD (visualized with anti-RpoD antibody) is shown as a loading control. Where indicated, strains expressed a Myc-tagged Ndk from the chromosomal *ndk* locus. (F) Western blots showing histidine phosphorylation of immunoprecipitated Ndk from wild-type and *ΔsixA E. coli.* Phosphorylated protein was visualized with anti-N1-phosphohistidine antibody. (G) A schematic of the reactions mediated by Ndk without and with SixA. The data shown in this figure are representative of at least three independent experiments. Strains used in the experiments are listed in Table S1.

To explore further the possibility that SixA regulates Ndk activity, we looked at the effects of perturbing SixA expression on phenotypes associated with Ndk-null strains. One such phenotype is resistance to azidothymidine (AZT), a nucleoside analog that causes DNA damage and inhibits *E. coli* growth.^40^ Chemical genetic screens indicate that an *ndk* deletion increases AZT resistance in *E. coli*.^10,11^ This likely indicates that Ndk plays an important role in the synthesis of AZT triphosphate, which is required for AZT incorporation into DNA. If SixA dephosphorylates the phosphohistidine intermediate of Ndk (pHis-Ndk), then SixA overexpression should lower Ndk activity and increase AZT resistance. Indeed, a multi-copy plasmid expressing SixA produced a similar improvement in *E. coli* growth in the presence of AZT as that of an *ndk* deletion (Figure 3A), whereas SixA overexpression did not improve the growth of a *Δndk* strain, consistent with SixA acting through Ndk. The effect of SixA overexpression was dependent on the phosphatase activity of SixA, as expression of a catalytically inactive SixA mutant^27,30^ did not improve the growth of wild type (Figure S1). We also tested the effect of deleting *sixA*, which should increase Ndk activity and hence increase AZT toxicity if AZT triphosphate levels are limiting. We found that in the presence of AZT, a *ΔsixA* strain grew more slowly than wild type, whereas deleting *sixA* did not affect the growth of a *Δndk* strain (Figure 3B), which is again consistent with SixA dephosphorylating Ndk.

Strains with an *ndk* deletion also have a mutator phenotype.^9^ The mechanism for this increased mutation rate has been proposed to stem from the dNTP pool imbalance in Ndk-null strains, but this explanation remains controversial.^4,9,41,42^ We found that plasmid overexpression of SixA increased the frequency of spontaneous rifampicin resistance, which is consistent with decreased Ndk activity (Figure 3C). The effect was smaller than that of an *ndk* deletion, in contrast with the case of AZT resistance, where the effects of *Δndk* and plasmid-expressed SixA were comparable (Figure 3A). This difference may indicate that, in contrast with NDPs, AZT diphosphate is a relatively poor substrate for *E. coli* Ndk that can be outcompeted by SixA.^43^

### SixA dephosphorylates Ndk in *vitro* and *in vivo*

To test directly whether SixA dephosphorylates pHis-Ndk, we performed dephosphorylation assays with purified proteins. After phosphorylating Ndk by incubating with ATP, excess ATP was removed with desalting columns, and purified SixA was added to the reaction product. The phosphorylation state of Ndk was assessed by western blotting with an anti-phosphohistidine antibody that is specific for phosphorylation at the N1 position of the imidazole ring.^7,25,44^ Addition of SixA eliminated this phosphohistidine signal, whereas a catalytically inactive SixA (SixA H8A) had no effect (Figure 3D). Since phosphohistidine is heat-labile, boiled samples were also included to confirm loss of the phosphohistidine signal. These results establish that pHis-Ndk can serve as a SixA substrate.

If SixA dephosphorylates pHis-Ndk *in vivo*, then deleting *sixA* should increase intracellular pHis-Ndk levels. To test this, we assayed Ndk phosphorylation in wild-type and *ΔsixA E. coli*. For these experiments, we used *E. coli* strains that expressed a Myc-tagged Ndk from the native *ndk* locus. Cell lysates were analyzed by Phos-tag SDS-PAGE to separate phosphorylated proteins from the faster-migrating unphosphorylated forms, followed by western blotting with an anti-Myc antibody.^45^ We found that the fraction of Ndk that was phosphorylated was higher in the *ΔsixA* strain, supporting a role for SixA in regulating Ndk (Figure 3E).

We also assessed intracellular Ndk phosphorylation through immunoprecipitation. Using the same Myc-tagged strain as above, we immunoprecipitated Ndk from wild-type and *ΔsixA E. coli* cell lysates and analyzed Ndk phosphorylation by western blotting with anti-phosphohistidine antibody. We observed a higher phosphohistidine signal of immunoprecipitated Ndk from *ΔsixA E. coli* as compared to wild type (Figure 3F), which is again consistent with SixA dephosphorylating pHis-Ndk *in vivo*.

### Excess nucleosides or nucleobases suppress the survival defect of Δ*sixA E. coli*

**Figure 4.**
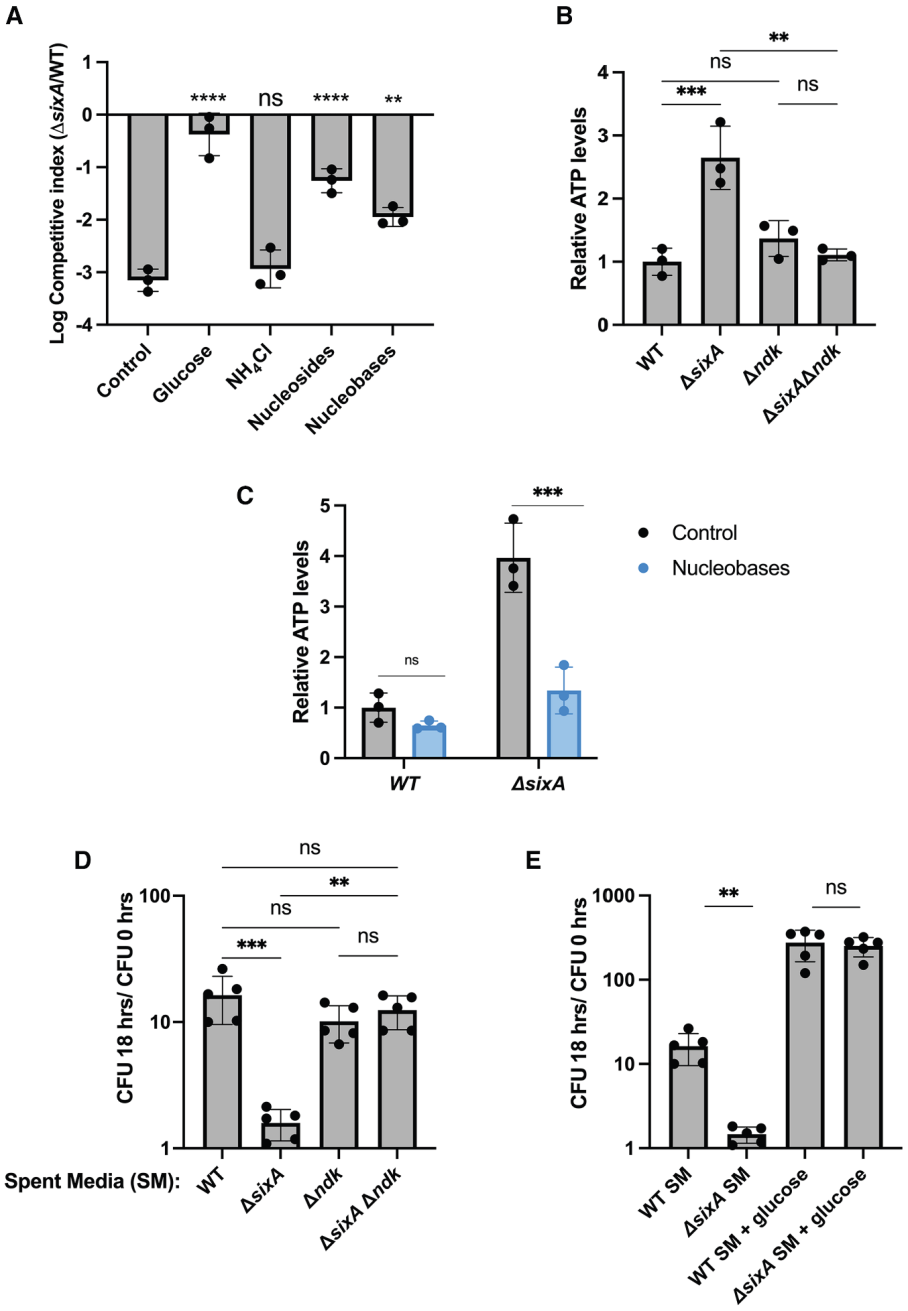
*ΔsixA E. coli* has altered physiology in stationary phase (A) Seven-day co-cultures of *ΔsixA* and wild-type *E. coli* in LB. The indicated compounds were added on day 4. Final concentrations were 0.2% glucose, 20 mM ammonium chloride, and 0.2 mM of an ACGU nucleoside or nucleobase mix. Error bars represent standard deviation. (B) Relative ATP levels of *ΔsixA*, *Δndk*, *ΔsixA Δndk*, and wild-type *E. coli* grown overnight in LB. ATP levels were measured with a luciferase assay and normalized by the average wild-type level. (C) Effect of nucleobase addition on relative ATP levels of *ΔsixA* and wild-type *E. coli*. After overnight growth in LB, 0.2 mM nucleobases were added and incubated for an additional 4 h before measuring ATP levels as in (B). (D) Overnight growth of wild-type *E. coli* in spent media prepared from 5-day cultures of *ΔsixA* or wild type in buffered LB. The ratios of CFU after 18 h to input CFU are shown. (E) Effect of glucose addition (to 0.2%) on overnight growth of wild-type *E. coli* in spent media (SM) as in (D). Symbols, filled bars, and error bars denote replicates, averages, and standard deviations, respectively. Statistical significance was calculated using Dunnett’s multiple comparison test (A), Tukey’s multiple comparison test (B and D), and unpaired *t* test (C and E). ns, **, ***, and **** represent *p* values > 0.05, ≤ 0.01, ≤ 0.001, and ≤ 0.0001, respectively. The Data shown in this figure are representative of at least three independent experiments. Strains used in the experiments are listed in Table S1.

The fact that a fitness defect of *ΔsixA E. coli* emerges in LB only after an extended time in stationary phase (Figure 1A) suggests that this phenotype depends on nutrient limitation. Glucose addition on day 4 restored the CFU of the *ΔsixA* strain to levels that were comparable to those of wild type (Figure 4A), consistent with carbon source availability playing a role. The addition of a nitrogen source, ammonium chloride, on the other hand, had no effect. Since increased expression of the nucleoside porin Tsx also rescued the fitness defect (Figures 2A and 2B), we tested the effect of adding a mixture of nucleosides on day 4. This partially suppressed the survival defect of the *ΔsixA* strain (Figure 4A). Once imported into the cell, nucleosides can provide a carbon source for *E. coli* through the degradation of their pentose moieties, or they can be salvaged to produce nucleotides.^37^ Nucleobases, on the other hand, can also be salvaged for nucleotide synthesis but cannot be used as carbon sources by *E. coli*.^37,46^ When we added a mixture of nucleobases on day 4 of stationary phase competitions, we found that the fitness defect of the *ΔsixA* strain was again partially suppressed (Figure 4A). These results suggest that alterations in nucleotide pools may contribute to the *ΔsixA* fitness defect in starvation conditions.

### Perturbed ATP levels in *ΔsixA E. coli*

SixA-mediated dephosphorylation of pHis-Ndk diverts a phosphoryl group from an NTP donor that, without SixA, would have been transferred to an NDP (Figure 3G). Therefore, SixA’s activity on Ndk could affect the NTP pool. To investigate this, we measured ATP levels in overnight cultures of *E. coli*. We focused on ATP because it is easily measured with a luciferase assay and also because it is likely one of the primary phosphoryl group donors to Ndk due to its high concentration in the cell compared to other NTPs.^47^ We found that ATP levels are significantly higher in *ΔsixA* compared to wild type and observed similar behavior in seven-day stationary phase cultures (Figures 4A and S2). A *ΔsixA Δndk* strain, on the other hand, showed ATP levels similar to wild type (Figure 4B). In the absence of Ndk, several enzymes—pyruvate kinase, succinyl-CoA synthetase, and adenylate kinase—have been suggested to play a compensatory role in phosphorylating NDPs,^4^ which may explain why the ATP levels of *Δndk* are similar to those of wild type. We also found that supplementation with nucleo-bases, a treatment that partially suppressed the fitness defect of *ΔsixA* (Figure 4A), reduced ATP levels of this strain (Figures 4C and S3).

### *E. coli* lacking *sixA* depletes nutrients more rapidly than wild type in monoculture

The above results suggest that Ndk regulation by SixA plays a critical role in *E. coli* metabolism under low-nutrient conditions. To investigate whether the loss of this regulation affects nutrient consumption, we assessed the capacity of spent media from wild-type and *ΔsixA E. coli* to support the growth of wild type. Spent medium from a five-day wild-type culture produced approximately a 10-fold increase in CFU after eighteen hours when inoculated with ~10^7^ CFU/mL of wild-type cells (Figure 4D). In contrast, spent medium from *ΔsixA* cells failed to produce any significant growth. In addition, the growth in spent medium from a culture of *ΔsixA Δndk* cells was similar to that of wild type, suggesting that the absence of growth in *ΔsixA* spent medium arises from the loss of the SixA-Ndk interaction. We also found that glucose supplementation produced comparable growth for spent media prepared from *ΔsixA* and wild-type cultures (Figure 4E). This suggests that the failure of the *ΔsixA* spent medium to support growth was not due to the presence of a growth-inhibiting factor but instead reflects the depletion of one or more nutrients.

## DISCUSSION

We have shown that in *E. coli*, the nucleoside diphosphate kinase Ndk is regulated by the phosphohistidine phosphatase SixA, and that eliminating this interaction by deleting *sixA* reduces *E. coli* fitness in long-term stationary phase. Ndk transfers the gamma phosphate from an NTP to an NDP. SixA inhibits this reaction by dephosphorylating the Ndk phosphohistidine intermediate, which has the effect of consuming an NTP without generating a new NTP (Figure 3G). Thus, deleting *sixA* could perturb the balance and total concentration of NTPs. Indeed, we found that in stationary-phase cultures, cellular ATP was significantly higher in the *ΔsixA* strain than in wild type, an effect that was dependent on *ndk* (Figure 4B). This is consistent with the mechanism described above, although we cannot rule out that the elevated ATP levels are due to other metabolic effects of increased Ndk phosphorylation in the *ΔsixA* strain. We also found that nucleobase supplementation lowered the ATP levels of *ΔsixA*. One possible explanation for this observation is that nucleobase import leads to increased Ndk-mediated phosphorylation of NDPs to NTPs, which would consume the major phosphoryl donor, ATP. However, it is also possible that the decrease in ATP arises from some other effect of nucleobase supplementation on cell metabolism in *ΔsixA.*

We found that wild-type *E. coli* did not grow in spent medium from a *ΔsixA* strain, in contrast with its ability to grow in wild-type spent medium. These results indicate that without SixA, *E. coli* depletes nutrients more rapidly in long-term stationary phase. The fact that adding nucleobases partially suppresses the *ΔsixA-*dependent fitness defect in long-term stationary phase suggests that the affected pathways involve nucleotide metabolism. This is further supported by our finding that deleting CytR and overexpressing Tsx both ameliorate the fitness defect. We hypothesize that this perturbed nucleotide metabolism lowers the fitness of the *ΔsixA* strain in starvation conditions. However, it is unlikely that the effect on fitness is solely due to increased nutrient depletion since, in competitions, a depletion of nutrients would also affect the survival of wild type.

At present, we do not know the nature of the metabolic dysfunction of *ΔsixA E. coli* and how it causes the fitness defect reported here. However, we note that perturbed nucleotide levels have been shown to affect bacterial survival in slow-growing or quiescent states in various contexts.^48-53^ Furthermore, beyond their roles as substrates in metabolic reactions, NTPs and their derivatives also act as signaling molecules regulating various biological functions. It might seem counterintuitive that a *ΔsixA* strain has a fitness defect when nutrients are scarce, despite having higher ATP levels in the early stages of stationary phase. However, high ATP could interfere with the physiological adaptations needed to survive extended periods of starvation. The combined activity of SixA and Ndk might function as a controlled futile cycle to fine-tune the ATP concentration. Indeed, there are many examples in which futile cycles and other apparently ‘‘wasteful’’ reactions are important for fitness.^53^

Previous studies have shown that SixA also dephosphorylates the phosphocarrier NPr,^30,32^ the glycolytic enzyme PfkA,^34^ and the sensor kinase ArcB (*in vitro)*.^27,28^ The three SixA targets identified to date do not share any obvious sequence or structural motifs around their sites of histidine phosphorylation, and it’s unclear what governs the specificity of SixA’s interactions with these proteins. A previous study noted that, based on the structures of SixA, NPr, and the ArcB HPt domain, structural rearrangements of SixA and/or NPr and ArcB would likely be required to account for SixA dephosphorylation of these proteins.^54^ An analogous situation appears to be the case for Ndk’s interaction with SixA. Ndk’s phosphorylated histidine is at the base of a cleft that is accessible to nucleotides, but does not appear to be accessible to the active-site histidine of SixA that catalyzes dephosphorylation. Similar observations have been made for the interaction between PGAM5 and NDPK-B.^54,55^ In addition, a recent study of *Mycobacterium tuberculosis* Ndk suggests there may be an equilibrium between the established Ndk structure and a more open conformation that might accommodate interactions between the phosphorylated histidine of Ndk and other proteins.^56,57^

SixA and PGAM5 are not the only phosphohistidine phosphatases that regulate nucleoside diphosphate kinase activity. In eukaryotes, PHPT-1/PHIP-1 regulates NDPK activity indirectly by dephosphorylating some kinase targets of NDPK.^58,59^ In addition, very recently, it was reported that Ndk from some *Mycobacterium* species is dephosphorylated by another phosphatase, MutT1.^60^

SixA and PGAM5 belong to the histidine phosphatase superfamily, a large set of enzymes that employ the transient phosphorylation of an active-site histidine for catalysis.^61^ Their phosphatase domains share limited sequence and structural homology that places them within the same branch of this superfamily, but they are nevertheless fairly disparate.^54^ In addition, PGAM5’s interaction with NDPK-B requires an N-terminal domain,^25^ which is missing from SixA^62^ (Figure S4). Interestingly, MutT1 is also in the same branch of the histidine phosphatase superfamily as SixA and PGAM5,^54^ but its activity against Ndk is through a Nudix hydrolase domain rather than its histidine phosphatase domain.^60^ It is thus striking that, despite these differences, SixA, PGAM-5, and MutT1 dephosphorylate nucleoside diphosphate kinases that are highly homologous both in amino acid sequence and in the structures of their monomers.^57^ Given the ubiquity of nucleoside diphosphate kinases and the prevalence of SixA orthologs across many bacterial phyla, it is likely that there are more examples of phosphohistidine phosphatases that target Ndk enzymes to modulate NTP pools and other cellular properties.

### Limitations of the study

While we showed that the SixA-Ndk interaction is important for *E. coli* survival during prolonged stationary phase, our understanding of the effect of this interaction on stationary phase physiology is incomplete. We observed an Ndk-dependent difference in ATP levels between wild type and Δ*sixA*, but we have not determined whether the defect observed during competitions is due to this ATP imbalance. In addition, we have not assessed whether the loss of SixA also affects the levels of other NTPs or NDPs, given that Ndk can use all NTPs/NDPs as substrates. At present, the mechanism by which altered nucleotide metabolism impacts bacterial fitness during starvation remains unclear. Moreover, while our spent media data (Figure 4) suggests differing nutrient consumption between wild-type and Δ*sixA,* the specific nutrients consumed, metabolic pathways involved, and their connection to NTP imbalance require further investigation.

## STAR★METHODS

### EXPERIMENTAL MODEL AND STUDY PARTICIPANT DETAILS

The bacterial strains used in this study are listed in the key resources table. Except where indicated otherwise, the growth medium was LB Miller Broth (10g Tryptone, 5g yeast extract and 10g NaCl/liter; Fisher Scientific-BP1426-500). Strains were grown at 37° C with aeration, except for certain strain construction steps in which plasmids with temperature-sensitive origins of replication were grown at 30° C. Where indicated, LB was buffered with 100 mM MOPS pH7. Antibiotics were used at the following final concentrations: 50 μg/mL kanamycin, 50 μg/mL ampicillin, 12.5 μg/mL chloramphenicol, 15 μg/mL tetracycline, 100 μg/mL rifampicin. Plasmids expressing proteins under the control of the *trc* promoter were not induced with IPTG (Isopropyl-β-D-thio-galactopyranoside); protein expression was from the basal activity of the *trc* promoter in pTrc99a. When indicated, nutrients were added to bacterial cultures at the following final concentrations: glucose 0.2%, ammonium chloride 20 mM, nucleoside mix (0.2 mM each of adenosine, cytidine, guanosine and uridine), or nucleobase mix (Teknova-M2103).

### METHOD DETAILS

#### Strain and plasmid construction

The P1vir phage transductions used to construct various strains are listed in the key resources table. Kanamycin resistance cassettes, which were flanked by FRT sites, were removed with Flp recombinase using pCP20.^68^ Gene deletions were confirmed by PCR.

A sequence encoding the Myc tag (EQKLISEED) was inserted at the end of the *ndk* gene by recombineering.^69^ Primers Ndk-myc-lred-FP and Ndk-myc-lred-RP were used to amplify a cassette consisting of the Myc-tag sequence ending with a stop codon followed by a kanamycin resistance flanked by FRT sites from BOP308^64^ genomic DNA. After electroporating the PCR product into MG1655/pKD46, recombinants were selected on kanamycin plates. The resulting *ndk-myc* FRT*-kan-*FRT sequence was moved into a clean MG1655 background as well as JES13 by P1vir phage transduction.

To construct the *tsx* expression plasmid (pARS6), primers F-BamHI-tsxRbs and R-HindIII-tsx-end were used to amplify the *tsx* gene from MG1655 genomic DNA. The PCR product was digested with HindIII and BamHI, and cloned into pTrc99a digested with same set of enzymes.

The Ndk-TEV-8XHis tag expression plasmid (pARS3) was constructed using two-fragment Gibson assembly with the 2X-Hifi DNA assembly kit (NEB-M5520A). The *ndk* sequence was amplified from MG1655 genomic DNA using the primers Ndk-gib-F and Ndk-gib-R, and the vector backbone was amplified from pJS65^32^ using pEKS-gib-F and pEKS-gib-R.

The insertions in the plasmids were verified by DNA sequencing.

#### Long-term competition assays

Strains for a competition were grown overnight at 37° C with aeration. Three microliters of each of the two overnight cultures were then added to a tube containing 3 mL LB medium and grown at 37° C on a roller drum for 7 days. For competitions between strains containing tetracycline-inducible fluorescent proteins, CFU were measured by spreading dilutions on LB-tetracycline plates as described previously.^67^ For competitions where one strain had a kanamycin resistance gene and the other strain was unmarked, CFU were determined by spot dilution on LB agar and LB-kanamycin agar. For strains containing ampicillin-resistant plasmids, the medium contained 50 μg/mL ampicillin during competition. For competitions with nutrient supplementation, the indicated nutrients were added on the fourth day of the competition. Competitive index was calculated using the following formula: $\text{Competitive Index}=[\mathrm{CFU}{\left(\text{day}\;7\right)}_{\text{strain}\;1}∕\mathrm{CFU}{\left(\text{day}\;0\right)}_{\text{strain}\;1}]∕[\mathrm{CFU}{\left(\text{day}\;7\right)}_{\text{strain}\;2}∕\mathrm{CFU}{\left(\text{day}\;0\right)}_{\text{strain}\;2}]$.

#### Long-term monocultures

LB was inoculated with a single colony of wild-type or mutant *E. coli* strains and grown overnight at 37° C with aeration. Five microliters of each culture were added to 5 mL of LB with 100 mM MOPS at pH 7 and grown at 37° C with aeration for 14 days. The culture volume was maintained at approximately 5 mL by adding sterile distilled water every second day. The CFU was measured by spot dilution on LB agar.

#### Genetic screen

Transposon mutagenesis of the strain ARS11 was done by electroporation of the plasmid pRL27, which contains a Tn5-derived mini-transposon.^70^ Transposon insertions were selected by growing overnight on LB-kanamycin agar; >18,000 mutants were obtained. The colonies were then scraped from the plate and resuspended in 1 mL LB. Five tubes, each containing 5 mL LB, were inoculated with 5 μL of this cell suspension, and the cultures were grown overnight. These overnight cultures were then competed against wild type (MAL190-2) as described above. After 7 days, the cultures were each spread on 50 μg/mL LB-kanamycin agar, and after overnight growth, the colonies from each competition were resuspended in 1 mL LB. To eliminate spontaneous mutants, the kanamycin insertions were transduced into clean backgrounds. To do this, for each cell suspension, 5 μL was inoculated into 5 mL LB and grown overnight. P1vir phage lysates were then prepared from these cultures and used to transduce kanamycin resistance into ARS11. Approximately 200 colonies were obtained from each of these transductions. These colonies were scraped from the plates, and another 5 competitions against MAL190-2 were performed as described above. After this second competition, kan resistance was again transduced into ARS11 as described above. Ten colonies from each of the five transductions were competed against MAL190-2 to identify suppressors. Kan resistance was then transduced into AFS14-3 and competed against MAL190-2 as described above to identify transposon insertions that increase the long-term fitness in a SixA^−^ but not in a SixA^+^ background. Genomic DNA sequences flanking the sites of transposon insertions in the suppressors were then identified as described previously.^71,72^

#### Growth in the presence of azidothymidine

Overnight cultures were diluted 1000-fold into LB containing either 20 ng/mL AZT with 50 μg/mL of ampicillin—for strains containing plasmids—or 10 ng/mL AZT (3′-Azido-3′ deoxythymidne, Alfa Aesar-J65127). 0.2mL of the diluted cultures were added to 96 well plates, and the growth was measured in a Bio-Tek Synergy-neo2 plate reader at 37° C with continuous double-orbital shaking. The optical density at 600nm was measured every 20 min for 24 h.

#### Rifampicin resistance assay

Strains were grown overnight in LB with 50 μg/mL ampicillin. One milliliter of each overnight culture was centrifuged, the pellet was resuspended in 200 μL LB and spread on LB-rifampicin agar to identify spontaneous mutants. The total CFU was measured by spot dilution on LB agar plates. Mutation frequencies were calculated as the ratio of rifampicin-resistant colonies to the total CFU.

#### Protein purification

BL21(DE3) cells with Ndk and SixA expression plasmids were grown to mid-exponential phase in LB with 50 μg/mL ampicillin. Protein expression was then induced by adding IPTG to 1 mM and the cultures were grown for an additional 3 to 4 h at 37° C with vigorous shaking. Cells were pelleted, resuspended in lysis buffer (50 mM NaH_2_PO_4_, 300 mM NaCl, 10 mM imidazole, 0.01 mg/mL DNAse I, 0.01 mg/mL RNAse A, 1mg/ml lysozyme, pH 8) and lysed by sonication on ice. Proteins were purified using Ni-NTA agarose (QIAGEN-30210) as per the manufacturer’s protocols. The 8X-His tag was removed by overnight incubation with His-tagged TEV protease (Sigma-T4455) at 4° C. Untagged protein was purified in the flow through after 1-h incubation with Ni-NTA agarose at 4 ° C. Protein was transferred into storage buffer (50 mM Tris HCl, 150 mM NaCl, 10% glycerol, pH 8) by dialysis and stored at −80° C. Protein yield was estimated with a Bradford assay (Bio-Rad Protein Assay-500-0006) using a BSA standard curve.

#### *In vitro* Ndk phosphorylation

To prepare phosphorylated Ndk, 100 μM ATP and 80 μM Ndk were incubated with reaction buffer (50 mM Tris HCl, 5 mM MgCl_2_, pH 8) at a final volume of 10 μL at 30° C for 20 min. After incubation, excess ATP was removed by passing through two desalting spin columns (Zeba 89882, Thermo Scientific). The phosphorylated Ndk was incubated with 150 μM SixA or SixA(H8A) or buffer for 30 min at 30° C in reaction buffer with 2 mM DTT. After 30 min the reactions were mixed with an equal volume of 2X loading dye (20% glycerol, 80 mM glycine, 10 mM Tris HCl, 0.01% bromophenol blue, pH 8.8, 100 mM DTT) and run on 4%–20% gradient Mini-PROTEAN TGX precast protein gels (Bio-Rad Laboratories-4561096) with running buffer (25 mM Tris, 192 mM glycine, 0.1% SDS, pH 8.3) at 4° C. Since phosphohistidine is heat labile, some samples were boiled as controls to eliminate the phosphohistidine signal. Protein transfer to 0.45 μm Immobilon-P PVDF membrane (Millipore-IPVH00010) was performed with an Invitrogen Power Blotter. Membrane was blocked with 5% milk in TBST (8 g NaCl, 0.38 g KCl, 3 g Tris base, 500 μL Tween 20 per liter at pH 7.4). The membrane was probed with anti-phosphohistidine antibody (Millipore Sigma-ZRB1330; RRID: AB_2868462), which was diluted in TBS (8 g NaCl, 0.38 g KCl, 3 g Tris base per liter at pH 7.4) to 0.6 μg/mL. Enhanced chemiluminescence (ECL) detection was performed with KwikQuant Ultra Digital-ECL Substrate (Kindle Biosciences-R1004), and membranes were imaged with a KwikQuant Imager (Kindle Biosciences).

#### *In vivo* Ndk phosphorylation assay

Cell pellets from 2 mL of overnight cultures in LB were dissolved in 1 mL 1X loading dye (10% glycerol, 40 mM glycine, 5 mM Tris HCl, 0.005% bromophenol blue, pH 8.8, 50 mM DTT) and lysed by sonication on ice. The proteins were separated by electrophoresis on 80 μM Phos-tag (NARD Institute Ltd.-AAL107) 15% polyacrylamide gel with chilled running buffer (25 mM Tris, 192 mM glycine, 0.1% SDS, pH 8.3) at 4° C. Protein Transfer was performed as described above, and membrane was probed with anti-Myc antibody (Santa Cruz Biotechnology-SC-40X; RRID: AB_627268) and anti-RpoD antibody (Santa Cruz Biotechnology-SC56768; RRID: AB_785576) for loading control. ECL detection was performed as described above.

#### Ndk-Myc immunoprecipitation

Immunoprecipitation of C-terminal Myc-tagged Ndk was performed using Anti-Myc magnetic beads (Thermo Scientific-88842; RRID: 2861398) following the manufacturer’s protocol. Cell pellets from 4 mL of overnight cultures in LB were resuspended in 1 mL c-Myc IP buffer 1 and lysed by sonication on ice. The binding reaction was performed at 4° C for 4 h. The final elution step was performed in 100 μL 50 mM sodium hydroxide. The eluted solution was mixed with 2X gel loading dye (20% glycerol, 80 mM glycine, 10 mM Tris HCl, 0.01% bromophenol blue, pH 8.8, 100 mM DTT). 20 μL of this solution was run on a 4%–20% gradient Mini-PROTEAN TGX precast protein gel (Bio-Rad Laboratories-4561096) with running buffer (25 mM Tris, 192 mM glycine, 0.1% SDS, pH 8.3) at 4° C. Protein Transfer was performed as described above. Membranes were probed with either anti-Myc antibody (Santa Cruz Biotechnology-SC-40X; RRID: AB_627268) or anti-phosphohistidine antibody (Millipore Sigma-ZRB1330; RRID: AB_2868462). ECL detection was performed as described above.

#### ATP assay

0.1 mL of overnight LB cultures were pelleted and washed once with phosphate buffer saline (PBS) (sodium chloride 9 g, sodium phosphate dibasic 0.795 g and potassium phosphate monobasic 0.144 g per liter), and resuspended in 1 mL distilled water. 100 μL of this suspension was used to measure the relative ATP concentrations using the Biotium ATP-Glo Bioluminometric cell viability assay kit. When indicated, saturated cultures were supplemented with an ACGU nucleobase mix and incubated for 4 h at 37° C with aeration prior to measuring ATP as described above. Luminescence was measured on a Bio-Tek Synergy-neo2 plate reader and normalized by OD_600_.

For ATP measurements of seven-day cultures (Figure S2), ATP levels were too low to be detected by the above method. We therefore prepared cell extracts from 1 mL of seven-day LB cultures using a previously described method.^73^ Cells were collected on 25 mm, 0.45 μm Polyethersulfone (PES) filters (Sterlitech-PES4525100) for the extraction. ATP in the extract was measured using the Biotium ATP-Glo Bioluminometric cell viability assay kit using appropriate dilutions.

#### Spent media

To prepare spent media, cultures were grown in LB (buffered with 100 mM MOPS pH 7) for 5 days at 37° C with aeration. Cultures were then centrifuged, and the supernatants were sterilized by passing through 0.2 μm filters that had been pre-rinsed with sterile distilled water. To assay growth in spent medium, 1 mL of a wild-type *E. coli* overnight culture was pelleted, washed once with PBS and resuspended in 1 mL of PBS. The cell suspension was diluted 1000-fold into the spent medium and grown at 37° C overnight with aeration. To test the effect of carbon source addition, 0.2% glucose was added to the spent medium along with the cells. The initial and final CFU after overnight growth were measured by spot dilution on LB agar.

### QUANTIFICATION AND STATISTICAL ANALYSIS

The statistical tests used are indicated in the figure legends. All statistical analyses were performed in GraphPad Prism.

## Supplementary Material

1

2

Supplemental information can be found online at https://doi.org/10.1016/j.celrep.2025.116813.

## Figures and Tables

**Table:** KEY RESOURCES TABLE

REAGENT or RESOURCE	SOURCE	IDENTIFIER
Antibodies		
anti-Myc antibody	Santa Cruz Biotechnology	Cat# SC-40X; RRID: AB_627268
Anti RpoD antibody	Santa Cruz Biotechnology	Cat# SC56768; RRID: AB_785576
Anti-Myc magnetic beads	Thermo Scientific	Cat# 88842; RRID: AB_2861398
anti-phosphohistidine antibody	Millipore Sigma	Cat# ZRB1330; RRID: AB_2868462
Bacterial strains		
*E. coli* MG1655: F- λ- *ilvG rfb-50 rph-1*	*E. coli* Genetic Stock Center	CGSC no. 7740
*E. coli* JW2337: Δ*sixA*::(FRT-*kan*-FRT)	Baba et al.^63^	JW2337
*E. coli* ARS162: MG1655 Δ*sixA*::(FRT-*kan*-FRT)	This study. P1vir(JW2337) X MG1655	N/A
*E. coli* JES13: MG1655 Δ*sixA*::(FRT)	Schulte and Goulian^30^	N/A
*E. coli* AFS14-3: MG1655 *attB_λ_* :pAS07(*cat tetR tetA-gfp*)	Goulian lab stock	N/A
*E. coli* MAL190-2: MG1655 *attB_λ_*:pML8(*cat tetR tetA-mcherry)*	Goulian lab stock	N/A
*E. coli* ARS11: MG1655 Δ*sixA*::(FRT) *attB_λ_* :(*cat tetR tetA-gfp*)	This study. P1vir(AFS14-3) X JES13	N/A
*E. coli* JES185: MG1655 Δ*npr*::(FRT-*kan*-FRT)	Schulte and Goulian^30^	N/A
*E. coli* JES186: MG1655 Δ*npr*::(FRT-*kan*-FRT) Δ*sixA*::(FRT)	Schulte and Goulian^30^	N/A
*E. coli* JW3905: Δ*cytR*::(FRT-*kan*-FRT)	Baba et al.^63^	JW3905
*E. coli* ARS60: MG1655 Δ*sixA*::(FRT) *attB_λ_* :(*cat tetR tetA-gfp*) Δ*cytR*::(FRT-*kan*-FRT)	This study. P1vir(JW3905) X ARS11	N/A
*E. coli* ARS61: MG1655 *attB_λ_* :(*cat tetR tetA-GFP*) Δ*cytR*::(FRT-*kan*-FRT)	This study. P1vir(JW3905) X AFS14-3	N/A
*E. coli* ARS63: MG1655 Δ*sixA*::(FRT) *attB_λ_* :(*cat tetR tetA-gfp*) Δ*cytR*::(FRT)	This study. pCP20 treated ARS60	N/A
*E. coli* JW0401: Δ*tsx*::(FRT-*kan*-FRT)	Baba et al.^63^	JW0401
*E. coli* ARS64: MG1655 Δ*sixA*::(FRT) *attB_λ_* :(*cat tetR tetA-gfp*) Δ*cytR*::(FRT) Δ*tsx*::(FRT-*kan*-FRT)	This study. P1vir(JW0401) X ARS63	N/A
*E. coli* ARS87: MG1655 *attB_λ_* :(*cat tetR tetA-GFP*) Δ*tsx*::(FRT-*kan*-FRT)	This study. P1vir(JW0401) X AFS14-3	N/A
*E. coli* ARS88: MG1655 Δ*sixA*::(FRT) *attB_λ_* :(*cat tetR tetA-gfp*) Δ*tsx*::(FRT-*kan*-FRT)	This study. P1vir(JW0401) X ARS11	N/A
*E. coli* JW2408: Δ*ndk*::(FRT-*kan*-FRT)	Baba et al.^63^	JW2408
*E. coli* JES191: MG1655 Δ*ndk*::(FRT-*kan*-FRT)	This study. P1vir(JW2408) X MG1655	N/A
*E. coli* JES192: MG1655 Δ*ndk*::(FRT-*kan*-FRT) Δ*sixA*::(FRT)	This study. P1vir(JW2408) X JES13	N/A
*E. coli* BOP308: MG1655 *arcA*::(*arcA-6X-myc* FRT-*kan*-FRT)	Cho et al.^64^	N/A
*E. coli* ARS130: MG1655 *ndk*::(*ndk-myc* FRT-*kan*-FRT)	This study. MG1655 pKD46 + (*myc* FRT-*Kan*-FRT)	N/A
*E. coli* ARS131: MG1655 *ndk*::(*ndk-myc* FRT-*kan*-FRT)	This study. P1vir(ARS130) X MG1655	N/A
*E. coli* ARS134: MG1655 *ndk*::(*ndk-myc* FRT-*kan*-FRT) Δ*sixA*::(FRT)	This study. P1vir(ARS130) X JES13	N/A
*E. coli* PIR2: F– *Δlac169 rpoS*(Am) *robA1 creC510 hsdR514 endA recA1 uidA*(Δ*MluI*):*pir*	Invitrogen	N/A
*E. coli* TOP10: F– *mcrA* Δ(*mrr-hsdRMS-mcrBC*) *endA1 recA1* φ80*lacZ*ΔM15 Δ*lacX74 araD139* Δ(*ara-leu*) 7697 *galU galK rpsL nupG*	Invitrogen	N/A
*E. coli* BL21(DE3): F– *ompT hsdSB* (*rB*– *mB*–) *gal dcm* (DE3)	Novagen	N/A
Chemicals, peptides, and recombinant proteins		
10X ACGU solution	Teknova	Cat#M2103
Adenosine	TCI	Cat#A0152
Cytidine	Sigma	Cat#C4654
Guanosine	Sigma	Cat#G6752
Uridine	TCI	Cat#U0020
AZT	Alfa Aesar	Cat#J65127
Ni-NTA agarose	Qiagen	Cat#30210
TEV protease	Sigma	Cat#T4455
Phos-tag Acrylamide	Nard	Cat#AAL-107
KwikQuant Ultra Digital-ECL Substrate	Kindle Biosciences	Cat#R1004
Polyethersulfone membrane	Sterlitech	Cat#PES4525100
Critical commercial assays		
Protein Assay kit	Bio-Rad Laboratories	Cat#500-0006
ATP-Glo Bioluminometric cell viability assay kit	Biotium	Cat#30020
2X-Hifi DNA assembly kit	NEB	Cat#M5520A
Oligonucleotides		
Oligos used for constructing strains	See Table S2	N/A
Recombinant DNA		
pTrc99a: *lacI*q, P*_trc_*, MCS from pUC18, *rrnB*(Ter),*bla*, ori pMB1	Amann et al.^65^	N/A
pEB52: pTrc99a with NcoI site removed	Lippa et al.^66^	N/A
pAS07: *ori*R6K*γ attP_λ_ cat tetR tetA-gfp*	Lasaro et al.^67^	N/A
pML8: *ori*R6K*γ attP_λ_ cat tetR tetA-mcherry*	Lasaro et al.^67^	N/A
pSixA (pJS17): pTrc99a P*_trc_-sixA*(H8A)	Schulte and Goulian^30^	N/A
pSixA (H8A) (pJS21): pTrc99a P*_trc_-sixA*(H8A)	Schulte and Goulian^30^	N/A
pTsx (pARS6): pEB52 P*_trc_-tsx*	This study	N/A
pARS3: pET-41 PT7*lac-ndk*-TEV-(His)8	This study	N/A
pJS65: pET-41 PT7*lac-ptsO*-TEV-(His)8	Schulte et al.^32^	N/A
pJS67: pET-41 PT7*lac-sixA*-TEV-(His)8	Schulte et al.^32^	N/A
pJS70: pET-41 PT7*lac-sixA*(H8A)-TEV-(His)8	Schulte et al.^32^	N/A
pCP20: λ*c*I857(ts), λ*p*R-FLP, *repA101*(ts), *oriR101*, *bla*, *cat*	Cherepanov and Wackernagel^68^	N/A
pKD46: *repA101*(ts), *oriR101*, *bla*, P_*araB*_-(*gam bet exo*)	Datsenko et al.^69^	N/A
pRL27: [ME *ori*R6K*γ kan oriT* ME] P_*tetA*_-*tnp*	Larsen et al.^70^	N/A
Other		
Zeba desalting spin columns	Thermo Scientific	Cat#89882
Mini-PROTEAN TGX precast protein gels	Bio-Rad Laboratories	Cat#4561096
Immobilon-P PVDF membrane	Millipore	Cat#IPVH00010

## Highlights

- The phosphatase SixA is required for *E. coli* fitness during stationary phase
- Nucleobase import rescues the growth defect of *sixA* deletion mutants
- SixA dephosphorylates nucleoside diphosphate kinase (Ndk) *in vivo* and *in vitro*
- Loss of Ndk dephosphorylation by SixA leads to a stationary phase fitness defect
