# Supplementary material for: A phosphohistidine phosphatase promotes starvation survival by dephosphorylating nucleoside diphosphate kinase
Source: Cell Rep. Author manuscript; Available in PMC 2026 Feb 25. (PMC12934123; doi:10.1016/j.celrep.2025.116813)
Supplement: 1 [file NIHMS2142892-supplement-1.pdf]

Cell Reports, Volume *45*

Supplemental information

A phosphohistidine phosphatase promotes starvation survival by dephosphorylating nucleoside diphosphate kinase

Akash R. Sinha and Mark Goulian

Supplemental Figures

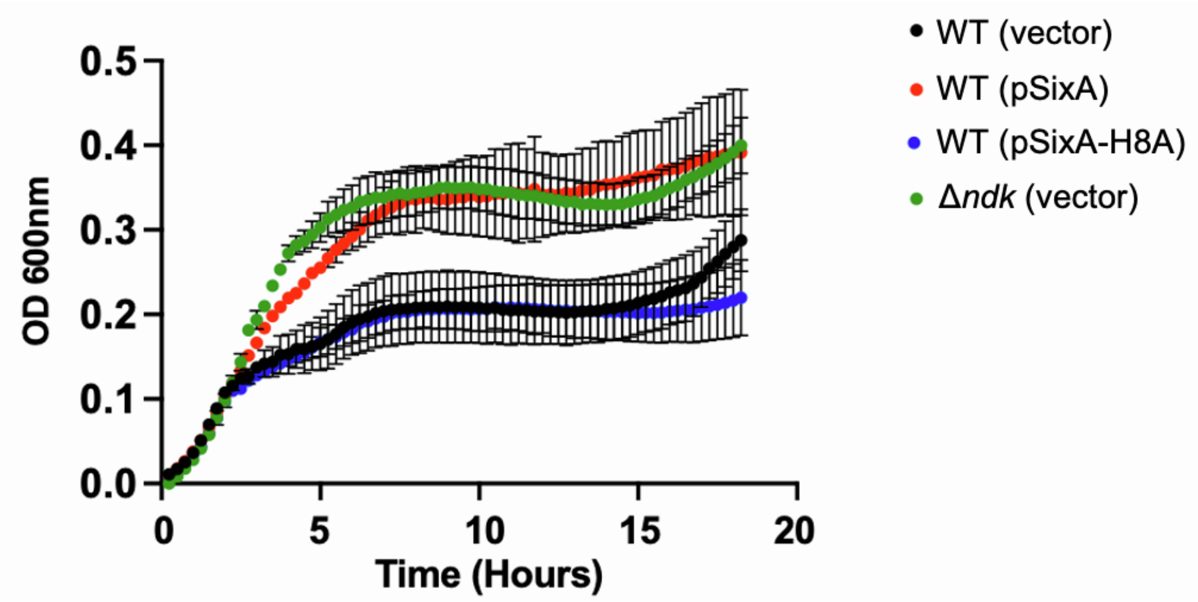

Figure S1. Effect of SixA overexpression on *ndk*-dependent phenotype

Growth curves of wild type or *Δndk E. coli* containing a plasmid expressing SixA (pSixA), catalytically inactive SixA (pSixA-H8A), or an empty vector in LB containing 20 ng/ml azidothymidine (AZT) and 50 µg/ml ampicillin. Symbols are the averages over three wells in a 96-well plate, and error bars are the standard deviations.

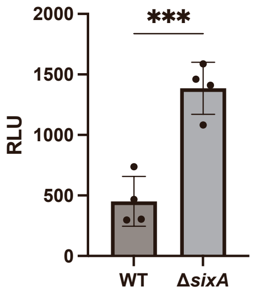

Figure S2. Relative ATP levels in nucleotide extracts from seven-day-old WT and *ΔsixA E. coli.*

Cell extracts were prepared from *E. coli* monocultures that were grown for seven days in LB (see Method Details), and ATP levels of the cell extracts were measured using a luciferase assay. Statistical significance was calculated using an unpaired t-test, and *** represents P values ≤ 0.001. Error bars denote standard deviations, symbols denote replicates, and filled bars denote averages.

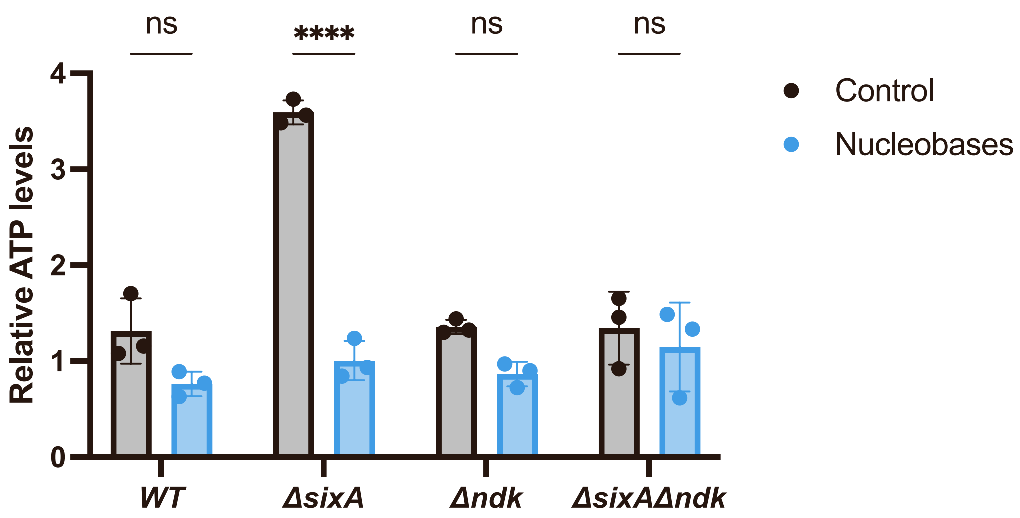

Figure S3. Effect of nucleobases on relative ATP levels in *E. coli.*

After overnight growth in LB, 0.2 mM nucleobases were added and incubated for an additional 4 hours before measuring ATP levels through a luciferase assay (see method details). Statistical significance was calculated using an unpaired t-test. ns and **** represent P values > 0.05, and ≤ 0.0001 respectively. Symbols, filled bars, and error bars denote replicates, averages, and standard deviations, respectively.

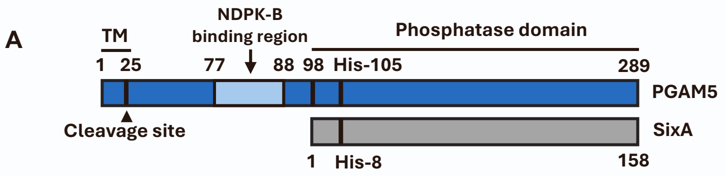

B

Cleavage site

```
PGAM5      MAFRQALQLAACGLAGGSAAVLFSAVAVGKPRAGGDAEPRPAEPPAWAGGARPGPGVWDP 60
SixA       ------------------------------------------------------------ 0


PGAM5      NWDRREPLSLINVRKRNVESGEEELASKLDHYKAKATRHIFLIRHSQYHVDGSLEKDRTL 120
SixA       -------------------------------------MQVFIMRHGDAALDAASDSVRPL 23
                                                 ::*::**.:  :*.: :. * *

PGAM5      TPLGREQAELTGLRLASLGLKFNKIVHSSMTRAIETTDIISR--HLPGVCKVSTDLLREG 178
SixA       TTNGCDESRLMANWLKGQKVEIERVLVSPFLRAEQTLEEVGDCLNLPSSAEV----LPEL 79
           *  * ::::.* .  * .  ::::::: * : ** :* : :.   :**. .:*    * *

PGAM5      APIEPDPPVSHWKPEAVQYYEDGARIEAAFRNYIHRADARQEEDSYEIFICHANVIRYIV 238
SixA       TPCGDVGLV---------------------SAYLQALT--NEGVASVLVISHLPLVGYLV 116
           :*      *                       *::     :*  :  :.*.*  :: *:*

PGAM5      CRALQFPPEGWLRLSLNNGSITHLVIR-PNGRVALRTLGDTGF---MPPDKITRS---   289
SixA       A-------------ELCPGETPPMFTTSAIASVTLDESGNGTFNWQMSPCNLKMAKAI   161
           .             .*  *.   :.     . *:*   *:  *   * * ::. :
```

Figure S4. An alignment of SixA with the PGAM5 L-isoform

Cytosolic PGAM5 L-isoform, produced by cleavage between residues 25 and 26 of PGAM5-L, dephosphorylates NDPK-B [1]. The active-site residues arginine and histidine of both proteins are shown in bold [2]. The sequence alignment was produced using the CLUSTAL O(1.2.4) multiple sequence alignment program [3].

Supplemental Tables

Table S1 : Strains used in the figures

**Figure**	**Strains**
1A	MG1655, ARS162
1B	MG1655, ARS162
1C	MG1655, ARS162, JES185, JES186
1D	ARS11/pTrc99a, ARS11/pJS17, ARS11/pJS21, MAL190-2/pTrc99a
2A	ARS11, ARS60, ARS61, ARS64, ARS87, ARS88, MAL190-2
2B	ARS11/pTrc99a, ARS11/pARS6, MAL190-2/pTrc99a, AFS14-3/pARS6
2C	MG1655, ARS162, JES191, JES192
2D	MG1655, ARS162, JES191, JES192
3A	MG1655/pTrc99a, MG1655/pJS17, JES191/pTrc99a, JES191/pJS17
3B	MG1655, ARS162, JES191, JES192
3C	MG1655/pTc99a, MG1655/pJS17, MG1655/pJS21, JES191/pTrc99a
3E	MG1655, ARS131, ARS134
3F	MG1655, ARS131, ARS134
4A	ARS11, MAL190
4B	MG1655, ARS162, JES191, JES192
4C	MG1655, ARS162
4D	MG1655, ARS162, JES191, JES192
4E	MG1655, ARS162

Table S2 : Oligos used in this study

Primer	Sequence	Resulting strain/ plasmid
F-BamHI-tsxRbs	CCACACAGGATCCTTCACTCCCGCAAGGGATT	pARS6
R-HindⅢ-tsxend	CCACACAAAGCTTCAATCAGAAATGCCGGGAA	pARS6
pEKS-gib-F	AAGTGTGCCCGCGCACCCGTGGTACCGAAAACCTGTATTTTCAGGGC	pARS3
pEKS-gib-R	GTACGTTCAATAGCcatTCATATGTATATCTCCTTCTTAAAG	pARS3
Ndk-gib-F	AAGGAGATATACATatgaatgGCTATTGAACGTACTTTTTCC	pARS3
Ndk-gib-R	TACAGGTTTTCGGTACCACGGGTGCGCGGGCACAC	pARS3
Ndk-Myc-lred-FP	GCGAAATCGCTTATTTCTTTGGCGAAGGCGAAGTGTGCCCGCGCACCCGTGAACAGAAACTGATTAGCGAAG	ARS130
Ndk-Myc-lred-RP	GCATTGTACAAATTCTGGCGCACGGATGCCACGTTTGCACGCGGCATTTATCCGTCGACCTGCAGTTCG	ARS130

References

1. Panda, S., et al., *Identification of PGAM5 as a Mammalian Protein Histidine Phosphatase that Plays a Central Role to Negatively Regulate CD4(+) T Cells.* Molecular Cell, 2016. **63**(3): p. 457-469.
2. Rigden, D.J., *The histidine phosphatase superfamily: structure and function*. The Biochemical Journal, 2008. **409**(2): p. 333-348.
3. Madeira, F., et al., *The EMBL-EBI Job Dispatcher sequence analysis tools framework in 2024*. Nucleic acids research, 2024. **52**(W1): p. W521-W525.
